# Improving Clinical Teaching Fellow Handover and Induction Preparedness: A Completed Closed-Loop Clinical Audit at Sandwell and West Birmingham Hospitals NHS Trust

**DOI:** 10.7759/cureus.108563

**Published:** 2026-05-09

**Authors:** Jawad Ahmad

**Affiliations:** 1 Undergraduate Education Department, Sandwell & West Birmingham Hospitals NHS Trust, West Bromwich, GBR

**Keywords:** clinical audit, clinical teaching fellow, handover, induction, medical education, quality improvement, sbar tool, standardisation

## Abstract

Background: Clinical teaching fellows (CTFs) provide continuity for undergraduate clinical education, including induction delivery, timetabling coordination, bedside/clinical skills teaching, and day-to-day liaison between students and clinical teams. At Sandwell and West Birmingham Hospitals NHS Trust, West Bromwich, England, the CTF team supports placements for Aston University and the University of Birmingham medical students across Years 3-5 (>150 students), making reliable cohort-to-cohort handover critical.

Methods: We conducted a closed-loop clinical audit of CTF handover package usability and perceived operational readiness using an anonymous Google Forms (Google LLC, Mountain View, CA, USA) questionnaire with two Likert-scale items (clarity of the handover document; ease of navigation, scale 1-5), four yes/no process measures (access details current; responsibilities clear; induction information sufficient; digital tools effective), and free-text comments. Baseline data reflected the 2024-2025 cohort’s received handover (8^th^ to 22^nd^ July 2025; n=6/6). Following a structured improvement package (formal handover presentation; rewritten and reorganised handover document; updated operational access information and signposting), re-audit data were collected from the incoming 2025-2026 cohort (4^th^ to 10^th^ August 2025; n=5/7; only five were able to attend the handover meeting due to prior work commitments). This was a local, closed-loop quality improvement clinical audit within a single NHS Trust.

Results: Mean ratings improved from 2.5/5 to 4.6/5 for document clarity and from 2.7/5 to 4.8/5 for ease of navigation. Process measures improved from 33% to 100% for access details, 17% to 100% for role clarity, 0% to 100% for induction sufficiency, and 83% to 100% for digital tool implementation.

Conclusions: A structured handover package was associated with substantial improvement in handover package usability and perceived operational readiness for incoming CTFs. Standardised annual handover (slide deck plus version-controlled document and checklist) is recommended to sustain improvements.

## Introduction

Handover is widely recognised as a vulnerable point for continuity and safety because responsibility and critical operational information are transferred between teams. UK handover guidance recommends that transfers should be structured and standardised, include an opportunity for questions, and be supported by reliable written information to reduce omissions and ambiguity [1,2]. Evidence from healthcare settings demonstrates that handoff communication failures are common and may be associated with adverse events, inefficiency, and avoidable rework [3].

Interventions that impose structure on handover have been associated with improved communication and patient-safety outcomes. The I-PASS (illness severity, patient summary, action list, situation awareness and contingency planning, and synthesis by the receiver) handoff programme, for example, was associated with reductions in medical errors and preventable adverse events in a multicentre study [4]. Similarly, structured communication tools such as SBAR (Situation, Background, Assessment, Recommendation) have been evaluated across settings, with systematic review evidence suggesting improvements in communication quality and patient-safety indicators when SBAR is implemented as part of broader systems change [5].

Although these frameworks are typically described for clinical handover, similar risks arise when educational and operational responsibility transfers between clinical teaching fellow (CTF) cohorts. CTFs often coordinate induction, timetables, and teaching delivery for large numbers of students, and the role can be challenging to start without clear guidance and early access to the necessary systems [6]. In parallel, literature on induction programmes for clinicians indicates that structured onboarding is commonly evaluated through perceived preparedness and confidence measures, supporting the relevance of user-reported outcomes when auditing readiness for practice [7].

At Sandwell and West Birmingham Hospitals NHS Trust, West Bromwich, England, the CTF team supports placements for Aston University and the University of Birmingham medical students across Years 3-5 (>150 students). During the 2024-2025 transition, incoming fellows reported gaps in access to information, role clarity, and induction guidance, prompting a completed closed-loop audit cycle to quantify baseline performance, implement improvements, and re-measure outcomes after the subsequent cohort-to-cohort handover.

Aims and audit standards

The primary aim was to improve the usability of the CTF handover package and the perceived operational readiness of incoming fellows to deliver induction and undergraduate teaching programmes. The audit focused on a handover document and associated operational processes such as access to information, role allocation, induction guidance, and use of digital tools.

Audit standards were adapted from UK best-practice guidance on structured handover and standardisation, including the need for clear allocation of responsibilities, reliable supporting information, and a structured handover process [1,2]. In line with clinical audit methodology, explicit thresholds were agreed locally prior to re-measurement [8].

Before the audit started, a “high-quality” handover package was defined as follows: the handover document should achieve an average score of at least 4/5 for both clarity and ease of use, and at least 90% of respondents should answer “yes” to each process measure, such as access to details being up-to-date and usability; roles and responsibilities being clearly defined across year groups and for both universities; induction information being sufficient; and digital tools being implemented effectively.

## Materials and methods

This project was conducted at Sandwell and West Birmingham Hospitals NHS Trust within the CTF team responsible for undergraduate placements for Aston University and the University of Birmingham. A closed-loop clinical audit design was used, measuring performance against explicit criteria, implementing change, and re-measuring to confirm improvement [8].

All eligible CTFs in the post were invited to participate, and participation was voluntary. Baseline respondents were the outgoing 2024-2025 CTF team, reflecting on the quality and usability of the handover they had received, with six of six eligible staff responding (n=6/6) between 8^th^ and 22^nd^ July 2025. The results of the initial measurements were presented at the local educational meeting as per the institutional audit guidelines, inciting the need for an intervention, which was subsequently introduced for the following 2025-2026 CTF cohort. Re-audit respondents were the incoming 2025-2026 cohort after delivery of the revised handover package, with five of seven eligible staff responding (n=5/7) between 4^th^ and 10^th^ August 2025; five attendees were present at the formal handover meeting, and two were unable to attend due to prior work commitments.

Data was collected via an anonymous Google Forms (Google LLC, Mountain View, CA, USA) questionnaire (Appendix A). The tool included two Likert-scale items (1-5) assessing the clarity and ease of navigation of the handover document; four yes-or-no process measures, including access to details, current and usable; responsibilities clarity of CTFs; induction information sufficiency; and digital tools effectiveness; and free-text fields for qualitative comments.

Descriptive statistics (means, medians, ranges, and proportions) were calculated for quantitative items, and free-text responses were summarised thematically. The improvement package was designed directly from baseline feedback and included a structured face-to-face handover supported by a standard slide deck with time for questions, a rewritten and reorganised handover document structured by year group and cohort with explicit responsibilities and practical guidance, and updated operational access information with clearer signposting to shared folders and tools.

This work evaluated an internal staff handover process and did not involve patients or patient records. The audit was registered with the Sandwell and West Birmingham Hospitals NHS Trust Audit/Clinical Governance Department (Audit ID 3297), and all questionnaire responses were anonymised at the point of collection.

Two CTF colleagues contributed to the design of the improvement package and reviewed the accuracy and clarity, while authorship responsibility remained with the corresponding author. Reporting was informed by established quality improvement reporting principles to enhance transparency and reproducibility [9].

## Results

Baseline survey responses were received from all six eligible members of the outgoing CTF team (n=6/6), and re-audit responses were received from five of seven eligible incoming fellows (n=5/7). Respondent roles are summarised in Table 1, and quantitative outcomes for Likert-scale measures and process measures are presented in Tables 2-3, respectively.

**Table 1:** Respondent roles at baseline and re-audit. CTF: clinical teaching fellow

Role	Baseline (n=6)	Re-audit (n=5)
Part-time CTF	1	0
Full-time CTF	2	3
Simulation fellow	2	2
Foundation Year 2 (F2, academic block)	1	0

**Table 2:** Change in Likert-scale outcomes between baseline and re-audit.

Measure	Baseline mean (median)	Baseline range	Re-audit mean (median)	Re-audit range
Document clarity (Likert scale 1–5)	2.5 (3)	1–3	4.6 (5)	3–5
Ease of navigation (Likert scale 1–5)	2.7 (3)	1–4	4.8 (5)	4–5

**Table 3:** Change in key handover process measures between baseline and re-audit.

Process measure	Baseline Yes (%)	Re-audit Yes (%)	Change (percentage points)
Access details current and usable	2/6 (33%)	5/5 (100%)	+67
Responsibilities clearly defined	1/6 (17%)	5/5 (100%)	+83
Induction information sufficient	0/6 (0%)	5/5 (100%)	+100
Digital tools implemented effectively	5/6 (83%)	5/5 (100%)	+17

**Figure 1:**
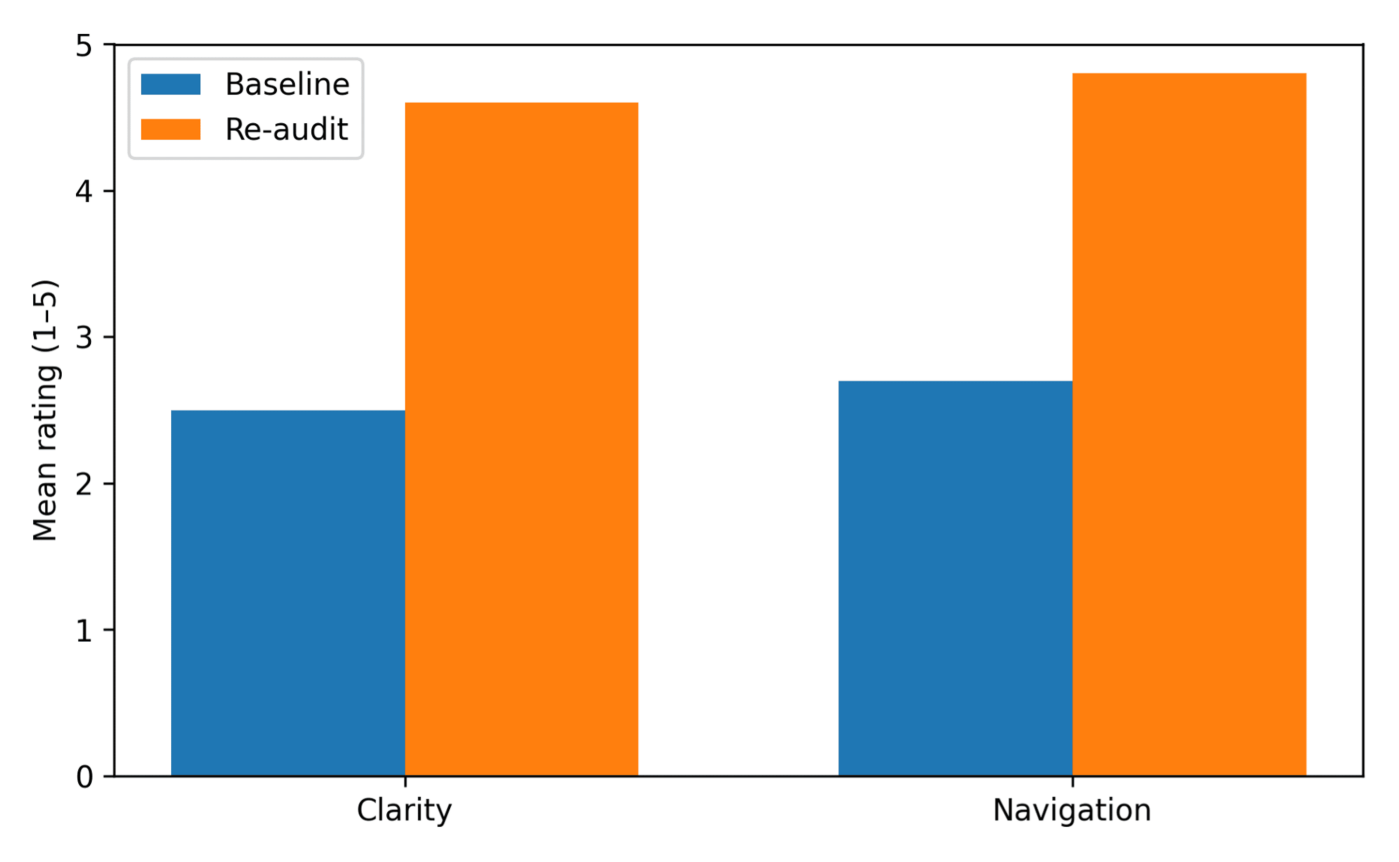
Mean Likert ratings (1–5) for clarity and navigation at baseline and after the handover intervention (re-audit). The plot is included to facilitate rapid comparison across cycles.

Likert-scale ratings for document clarity and ease of navigation improved substantially following implementation of the revised handover package. Mean clarity increased from 2.5 to 4.6 and mean navigation from 2.7 to 4.8, as shown in Table 2 and illustrated in Figure 1.

Likert items used a 1-5 scale, where 1 indicated very poor, and 5 indicated excellent. Higher scores represent better perceived usability of the handover document.

**Figure 2:**
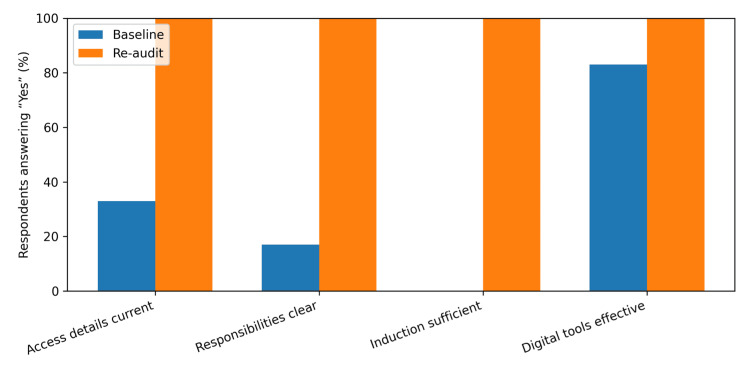
Percentage of respondents answering “Yes” to key handover process measures at baseline and re-audit. The plot highlights absolute improvements across all four measures.

All four process measures improved to 100% affirmative responses in the re-audit. The largest absolute improvement was observed for induction information sufficiency, increasing from 0% (0/6) at baseline to 100% (5/5) at re-audit, with other changes summarised in Table 3 and visualised in Figure 2.

Qualitative comments at baseline emphasised incomplete or outdated access information, insufficiently explicit allocation of responsibilities across year groups and the two university cohorts, limited practical detail for induction delivery, and variable signposting to existing digital resources. In the re-audit, no substantive new issues were raised, and respondents reported that key points were covered thoroughly with minimal need for further clarification.

Key findings

The re-audit met all prespecified thresholds for document usability and process reliability, including mean Likert ratings above 4/5 and 100% affirmative responses for each process measure. These findings indicate a marked improvement in perceived operational readiness following implementation of a structured handover package, while recognising that outcomes were self-reported and measured immediately after the intervention.

Figures 1-2 provide visual summaries of the quantitative findings described above. They are included for completeness and to support the interpretation of the changes between cycles.

## Discussion

Marked improvements in perceived usability and operational readiness followed the introduction of a standardised handover package. Rather than suggesting that a single element drove the change, the results are more plausibly explained by a bundle effect: consistent verbal handover (presentation), clearer written guidance (restructured document), and removal of predictable friction points (updated access information and signposting). This aligns with established handover principles, which emphasise standardisation, reliable supporting information, and clear allocation of responsibilities to reduce avoidable variation and prevent recurrent “start-of-rotation” problems [1-4].

Although most published guidance on handover is written for clinical environments, the same principles apply to educational and operational handover. Undergraduate teaching programmes rely on smooth continuity of core processes, including induction delivery, timetable management, agreed communication channels, and clear escalation pathways. When these processes are not effectively transferred between cohorts, significant time is spent rediscovering practical details and rebuilding local knowledge, rather than delivering teaching. A standardised handover package that is centred on a controlled document, a consistent PowerPoint slide, and a simple checklist is, therefore, likely to be effective because it turns informal, “in people’s heads” knowledge into clear, reproducible steps and reduces reliance on memory or ad hoc messaging [1-4].

The approach is potentially transferable to other education teams and rotational roles across NHS trusts. A practical model is an annual, protected handover meeting using a fixed agenda, supported by a single “source of truth” document with named ownership and a scheduled review date. To strengthen future cycles, objective endpoints could be added alongside staff-reported measures, such as the number of access issues logged in the first four weeks, time to independent induction delivery, or frequency of timetable errors requiring correction. This would allow triangulation between perceived readiness and measurable operational performance [5,6].

Several limitations should be noted. The sample size was small, and responses were self-reported, which introduces a risk of response and social desirability bias. Baseline responses reflected an earlier handover, so recall bias is possible. Additionally, the re-audit respondents were not identical to the baseline group, and only five of seven incoming fellows completed the post-handover questionnaire, introducing the potential for response bias if non-responders held different views. Furthermore, the re-audit occurred soon after the intervention, so a longer follow-up would be required to confirm durability beyond the immediate transition period. Despite these limitations, the audit cycle was completed against prespecified criteria, and the findings support continuation of a standardised annual handover process [5].

## Conclusions

A structured handover package for incoming CTFs, supported by a standard presentation and a revised, controlled handover document, was associated with improved perceived usability and self-reported operational readiness within a single NHS trust. Improvements were seen across both usability ratings and practical process measures, suggesting that common friction points, such as unclear responsibilities and incomplete access to information, can be reduced by standardising the handover approach. Although the project evaluated staff perceptions rather than objective operational outcomes, the findings support continuation of a consistent, repeatable handover format for CTF rotations.

To sustain these gains, an annual protected handover meeting is recommended, supported by a standardised slide deck, a checklist, and a single maintained “source of truth” document with named ownership and scheduled review. Future audit cycles could strengthen the evidence base by adding objective endpoints, such as the number of access issues logged in the first four weeks or the frequency of timetable errors requiring correction. Longer-term follow-up across subsequent cohorts would also help confirm the durability of the improvements and reduce the risk of regression over time. Overall, standardising educational handover is a feasible and low-cost intervention that may support smoother transitions for teams managing undergraduate teaching roles.

## Appendices

Appendix A

CTF Handover Audit Questionnaires Sandwell and West Birmingham Hospitals NHS Trust

Pre-handover (Baseline) questionnaire

1.     Please state your role.

2.     How clear was the information in the handover document for each academic year (Y3-Y5 + Simulation)?

3.     How easy was the handover document to navigate for each academic year (Y3-Y5 + Simulation)?

4.     Were login credentials, door codes, and equipment access details current, accurate, and easy to use?

5.     If you answered NO, could you tell us why?

6.     Did the document clearly define each CTF’s responsibilities and expected tasks for each year group and placement setting (UoB & Aston)?

7.     If you answered NO to the above question, could you tell us why?

8.     Was the induction information for each cohort (UoB, Aston) comprehensive and sufficient to run a smooth onboarding process?

9.     If you answered NO to the above question, could you tell us why?

10.  Were tools like SharePoint, Google Drive, SignUpGenius, and digital calendars implemented effectively to streamline planning and delivery?

11.  If you answered NO to the above question, could you tell us why?

12.  Are there any improvements or communication gaps which you would like to address for the future?

Post-handover (Re-audit) questionnaire

1.     Please state your role.

2.     How clear was the information in the handover document for each academic year (Y3-Y5 + Simulation)?

3.     How easy was the handover document to navigate for each academic year (Y3-Y5 + Simulation)?

4.     Were login credentials, door codes, and equipment access details current, accurate, and easy to use?

5.     If you answered NO, could you tell us why?

6.     Did the document clearly define each CTF’s responsibilities and expected tasks for each year group and placement setting (UoB & Aston)?

7.     If you answered NO to the above question, could you tell us why?

8.     Was the induction information for each cohort (UoB, Aston) comprehensive and sufficient to run a smooth onboarding process?

9.     If you answered NO to the above question, could you tell us why?

10.  Were tools like SharePoint, Google Drive, SignUpGenius, and digital calendars implemented effectively to streamline planning and delivery?

11.  If you answered NO to the above question, could you tell us why?

12.  Are there any improvements or communication gaps which you would like to address for the future?

Note: Both cycles used the same questionnaire items to enable direct comparison of responses.
